# Heat source modeling, penetration analysis and parametric optimization of super spray MAG welding

**DOI:** 10.1038/s41598-023-36505-6

**Published:** 2023-06-09

**Authors:** Zhifeng Li, Yimin Xia

**Affiliations:** 1 College of Mechanical and Electrical Engineering, Central South University, Changsha, 410083 China; 2 State Key Laboratory of High Performance Complex Manufacturing, Central South University, Changsha, 410083 China

**Keywords:** Mechanical engineering, Metals and alloys

## Abstract

Main drives, cutterheads and other critical components of tunnel shield machines require welding with thick plates that leave roots over 5 mm. Full penetration welds cannot be achieved by conventional Pulsed MAG welding methods. This article introduces Super Spray MAG Welding technology and investigates its penetrating regularities and mechanisms through high-speed camera images, finite element simulation, and microstructural analysis. An optimal welding procedure was generated using a combination of Genetic Algorithm and Back Propagation Neural Network. The data show that Super Spray MAG arc exhibits greater concentration and stability than traditional MAG arc, marking its strong qualities in emitting high-energy beams. The morphological solidification pattern of the molten pool closely matches the FEM simulation results of the composite Gaussian surface heat source model and peak linear attenuation Gaussian cylinder heat source. The welding current mainly affects the penetration of the weld, followed by the extension of the wire, and lastly the welding speed. Increasing the welding current can transition droplet transfer from globular to spray, as well as alter microstructure development and mechanical characteristics. Suggested parameters for penetrating the 5 mm root were put forward. The BPNN-GA model established can effectively predict weld formation, and points out the optimal welding parameters.

## Introduction

As tunnel construction technology advances, the size of tunnel boring machines (TBMs) continues to grow and the demand for greater load capacity increases. Key bearing components such as main drives and cutterheads bear large vibration loads during operation^1–3^. To ensure product dependability, T-joints are typically constructed using thick plates measuring at least 40 mm for both horizontal and vertical welding. In addition, more than 40% of full-penetration welds undergo initial-class ultrasonic testing. Compared to alternative methods such as TIG welding and submerged arc welding, MAG welding is frequently used in the production of TBM components because of its stable process, controllability, simple equipment, versatility, dependable joint quality, and high welding efficiency. Conventional MAG welding, due to its low welding current and arc pressure, faces difficulties in achieving full penetration of thick plates with root height exceeding 5 mm, and may lead to weld cracks, poor formation, and root defects. As a result, the rate of detection of qualified defects is low. Several techniques for optimizing the MAG welding process have been suggested, including raising the welding current, employing double-layer gas shielding, enhancing ultrasonic support, changing the properties of the joint shape, and so on^4–10^, but the arc energy is still low. Through a lot of industrial experiments, it has been found that MAG welding transitions into spray transfer when the welding current reaches about 400 A to properly penetrate the root height of 5 mm. As the temperature rises, the affected area expands, resulting in a more coarse grain structure and reduced mechanical properties^11–13^. Various post-weld heat treatments have been suggested to enhance penetration by transforming the microstructure^14, 15^. However, these treatments may affect local areas such as grain boundary precipitates, which can stabilize the microstructure and alleviate welding stress rather than promote rapid penetration. Several methods for optimizing the heat source, such as hybrid TIG/MAG welding and hybrid multi-wire MAG welding, have been proposed to improve droplet deposition rates^16–18^. However, these methods do not significantly increase the energy density at the welding site or enhance penetration depth. Conversely, the adaptability to grooves is reduced due to larger welding gun heads. Furthermore, high-energy beams like lasers and plasma are proposed as potential enhancements for increasing energy density^19–23^. But laser welding and plasma welding require highly precise assembly precision, have low energy efficiency, low cladding efficiency, and high equipment costs, making them unsuitable for producing large structural components.

This paper introduces a new Super Spray MAG welding technology. The welding arc is digitally controlled to produce a strong, focused, consistent beam. With removing the use of pulses, Super Spray MAG welding achieves a consistent and dependable arc to enhance droplet transfer frequency. However, it requires strict welding arc control to limit welding heat input. If the heat input is insufficient, it is difficult to penetrate the root. If the heat input is too large, it is easy to cause problems such as coarse grains, root defects, decline of mechanical properties, etc.^24, 25^. This paper presents an analysis of the heat flow distribution in Super Spray MAG Welding using FEM simulation. A combined heat source model is also proposed. The deep penetration phenomenon was investigated by analyzing spray arcs, melted droplets, microstructure, and joint properties. In addition, the combination of Back Propagation Neural Network and Genetic Algorithm was used to predict weld penetration and optimize process parameters. The research on Super Spray MAG Welding has provided valuable insights into heat source analysis, penetration regularities, and mechanisms. The stable and deep penetrating weld parameters obtained from this study can be used as a guide for welding thick plates in construction machinery.

## Experimental work

### Welding materials

**Figure 1:**
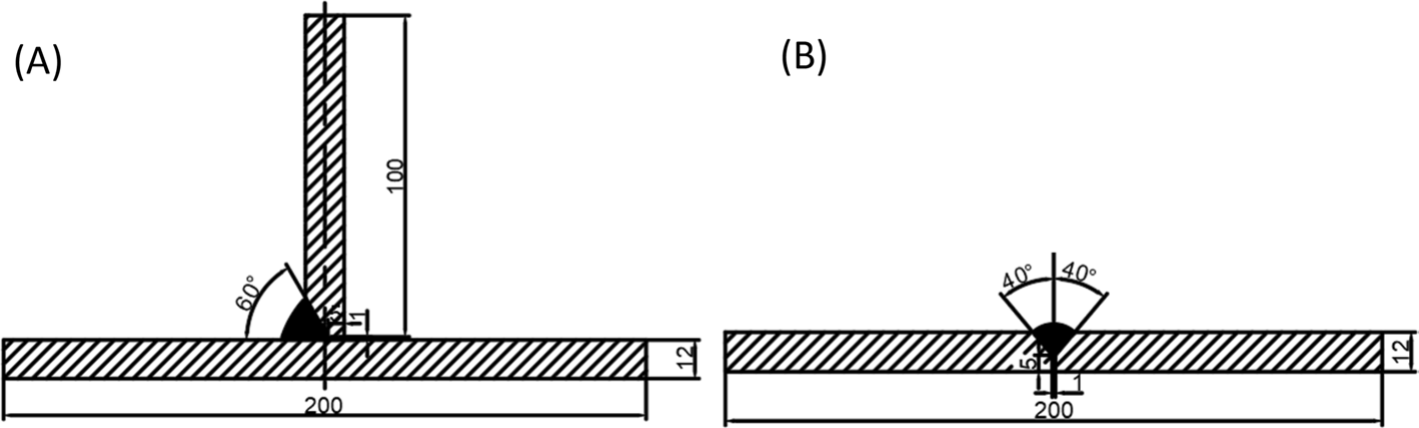
Structure and dimensions of: (**A**) T-joints, and (**B**) butt joints for welding tests.

Q355 steel plates were used as welding workpieces. The “V” groove weld joints with a 1 mm gap as shown in Fig. 1 were prepared with ER50-6 filler wires (DIN SG2). The filler wires were 1.2 mm in diameter. 1.2 mm. The chemical composition of the welded workpieces and filler wire is given in Table 1. Shielding gas was a mixture of 80% Ar + 20% CO_2_ with a flow rate of 15 L min^−1^.

**Table 1:** Composition of base metal and welding wire (wt.%).

Materials	C	Mn	Si	P	S	Ni	Cu	Fe
Q355	0.18	1.24	0.24	0.013	0.002	0.04	0.08	Balance
ER50-6	0.06–0.14	1.60–1.85	0.8–1.15	≤ 0.025	≤ 0.025	≤ 0.15	≤ 0.35	Balance

### Welding tests

All tests use robotic welding to ensure stability. The welding heat source was Qineo Pulse 600 Inverter. For each test, the groove shall be welded in one pass and free cooling down. The welding gun is located on the center line of the corner bisection of the joints and moves evenly perpendicular to the intersection line of the two vertical plates. The welding parameters are shown in Tables 2, 3. This experiment set up a group of orthogonal experiments to compare parameters, and a group of random experiments for BPNN prediction of weld microstructure and performance.

**Table 2:** Orthogonal experiment table for welding tests.

Level	Welding current/A A	Welding speed/(cm min^−1^) B	Wire extension/mm C
1	250	30	10
2	280	35	12
3	310	40	14
4	340	45	16
5	370	50	18

**Table 3:** Sample numbers and parameters in orthogonal welding tests.

Sample No	Welding arc	Welding current /A	Welding speed /(cm min^−1)^	Wire extension /mm
1	Super Spray MAG	250	30	10
2	Super Spray MAG	250	35	12
3	Super Spray MAG	250	40	14
4	Super Spray MAG	250	45	16
5	Super Spray MAG	250	50	18
6	Super Spray MAG	280	30	12
7	Super Spray MAG	280	35	14
8	Super Spray MAG	280	40	16
9	Super Spray MAG	280	45	18
10	Super Spray MAG	280	50	10
11	Super Spray MAG	310	30	14
12	Super Spray MAG	310	35	16
13	Super Spray MAG	310	40	18
14	Super Spray MAG	310	45	10
15	Super Spray MAG	310	50	12
16	Super Spray MAG	340	30	16
17	Super Spray MAG	340	35	18
18	Super Spray MAG	340	40	10
19	Super Spray MAG	340	45	12
20	Super Spray MAG	340	50	14
21	Super Spray MAG	370	30	18
22	Super Spray MAG	370	35	10
23	Super Spray MAG	370	40	12
24	Super Spray MAG	370	45	14
25	Super Spray MAG	370	50	16
26	Conventional Pulsed MAG	280	35	16

As shown in Table 2, the orthogonal test is set L_25_ (3^5^), i.e. three factors, five levels and a total of 25 trials, to compare and evaluate the impact of different factors. The corresponding sample number is shown in Table 3. Comparative parameters for Sample 26 were inherited from conventional MAG welding practices for steel plates below 40 mm. In addition, a groups of random experiments as shown in Table 4 were supplied to meet the sample size requirement of BPNN calculation. The extension of the wire sticking out of the nozzle is almost equal to the distance between the contact tip to work at the beginning. And it is set by trimming the length of the welding wire before arcing. Due to static characteristics of welding arc^26^, welding voltage is almost linearly determined by the current which can expressed as U = 0.04I + 20 ± 2 (V).

**Table 4:** Sample numbers and parameters in random welding tests.

Sample No	Welding arc	Welding current /A	Welding speed /(cm min−^-1)^	Wire extension /mm
27	Super Spray MAG	326	35	15
28	Super Spray MAG	370	38	12
29	Super Spray MAG	329	42	11
30	Super Spray MAG	356	50	15
31	Super Spray MAG	340	30	14
32	Super Spray MAG	334	40	14
33	Super Spray MAG	326	50	15
34	Super Spray MAG	275	37	17
35	Super Spray MAG	297	34	18
36	Super Spray MAG	304	42	11
37	Super Spray MAG	317	37	16
38	Super Spray MAG	355	46	16
39	Super Spray MAG	282	30	15
40	Super Spray MAG	356	47	16
41	Super Spray MAG	323	38	11
42	Super Spray MAG	264	44	18
43	Super Spray MAG	270	36	13
44	Super Spray MAG	337	31	17
45	Super Spray MAG	295	32	17
46	Super Spray MAG	326	46	16

### Characterization

**Figure 2:**
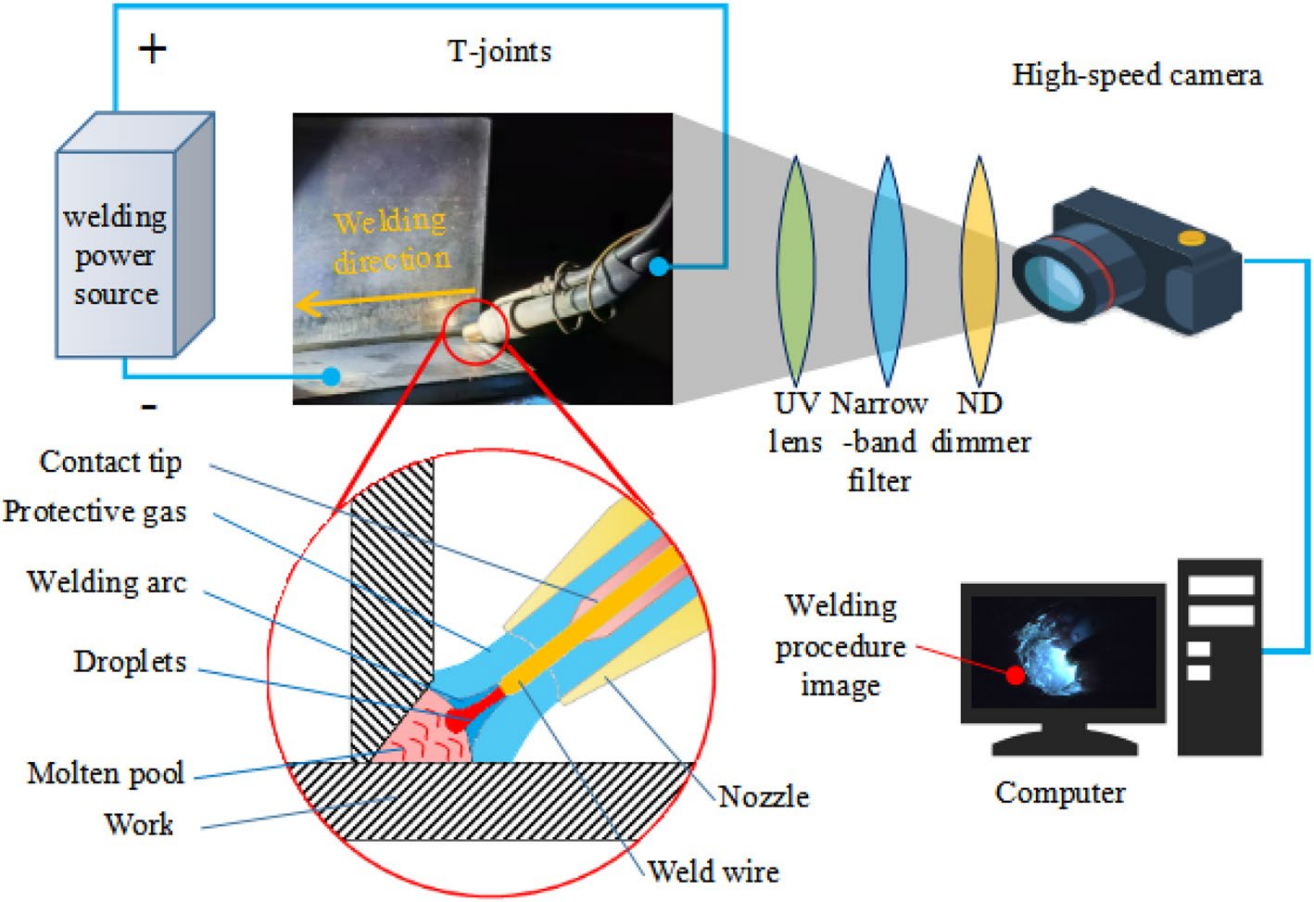
Schematic to capture images of welding procedures.

To analyze heat source properties, a finite element method was applied to simulate the heat flow distribution of welding arc. Meanwhile, a high-speed image acquisition system was established to capture images of welding procedures as shown in Fig. 2. The acquisition system included a computer, a Photron Limited SA1.1 high-speed camera and composite filter lenses. The filter lenses were composed of a high transparency UV lens, 800 nm narrow-band filter and a ND dimmer. The arc shape and droplet transfer process were captured by 8000 frames per-second and a shutter speed of 1/20,000 s.

To observe the microstructure of welds, optical microscopy (OM) was adopted. Before observation, the specimen was polished and viewed 100× under an optical microscope. No discernible scratches were identified. Use degreased cotton dipped in 4% nitric acid alcohol solution to wipe and corrode for 10–20 s.

To assess weld quality, Brinell hardness was estimated for each part of the joints. To verify optimal parameters, mechanical tests and ultrasonic flaw detection were performed after the groove had been filled by conventional MAG welding. According to EN ISO 15614 S*pecification and qualification of welding procedures for metallic materials—Welding procedure test*, mechanical tests should contain tensile, impact, and bending tests. The tensile test was performed at room temperature using the MTS-810 electrohydraulic servo material test system. The tensile strength of the joints must exceed that of the base metal. Impact test was performed using the CEAST9350 microcomputer controlled metal pendulum impact test machine. The minimum impact absorption energy of three standard specimens in each region of welded steel joints shall be above 20 J. It is acceptable for a specimen to have a slightly lower energy value, not falling below 70% of the specified minimum. The MTS-810 electrohydraulic servo material testing machine was also used for bending test. The center diameter for bending was d = 3a (with “a” being the thickness of the samples) and the bending angle was 180°. There should be no visible defects exceeding 3 mm in any direction. Ultrasonic testing was used to assess the integrity of welded joints in accordance with EN ISO 17640 and EN ISO 11666.

## Results and discussion

### Penetration simulation and heat source analysis

**Figure 3:**
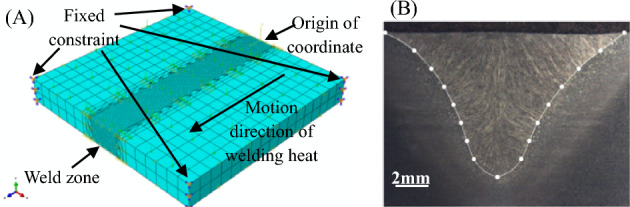
Welding heat source analysis: (**A**) CAE model, and (**B**) cross-section of Sample 11 butt joint.

During the welding process, the filler wire is heated until it melts into tiny droplets. These droplets then transfer to the base metal, forming a molten pool that cools down to create a weld seam. Thus, the configuration and energy dispersion of the welding arc dictate the droplet transfer mode and the ultimate microstructure and mechanical properties of joints. The section above the melting point is separated and analyzed against the real weld to examine the appropriateness of various heat source models. The finite element model for butt joints in welding has dimensions of 100 mm × 100 mm × 12 mm, as illustrated in Fig. 3A. The central weld area has C3D8 cell grids measuring 1 mm × 1 mm × 1 mm. The welding heat source moves in the positive Z direction and the model’s six degrees of freedom are restricted at its four corners. The predefined temperature field of the model is 20 ℃. The cross-sectional data of the butt joint weld for Sample 11 with a well-formed weld were collected, as depicted in Fig. 3B, indicating a weld width of 14.35 mm and a penetration depth of 8.63 mm. The welding parameters are as follows: welding current of 310A, welding speed of 30 cm min^−1^, wire extension of 14 mm, and a heat efficiency of 0.8^27^.

Table 5 shows the varying physical parameters of base steel at different temperatures. Through the linear interpolation method, thermophysical parameters are segmented into piecewise linear forms and used in CAE calculation.

**Table 5:** Thermophysical properties of Q355 at different temperatures.

Temperature/℃	Density /(kg m^−3^)	Specific heat capacity /(J kg^−1^ ℃^−1^)	Thermal conductivity /(W m^−1^ ℃^−1^)	Elastic modulus /GPa	Yield strength /MPa	Poisson ratio	Coefficient of linear expansion × 10^–6^/℃^−1^
0	7872	450	51.9	210	350	0.27	6.12
20	7866	458	51.74	207	345	0.28	6.17
100	7845	494	51.1	203	326	0.31	8.31
200	7816	526	49.0	197	302	0.32	10.1
300	7740	566	46.1	183	278	0.33	12.3
400	7733	615	42.7	173	229	0.35	13.2
500	7711	684	39.4	150	179	0.37	13.71
1000	7578	779	27.2	63	11	0.47	16.16
1500	7552	400	29.7	10	5	0.49	18.16

**Figure 4:**
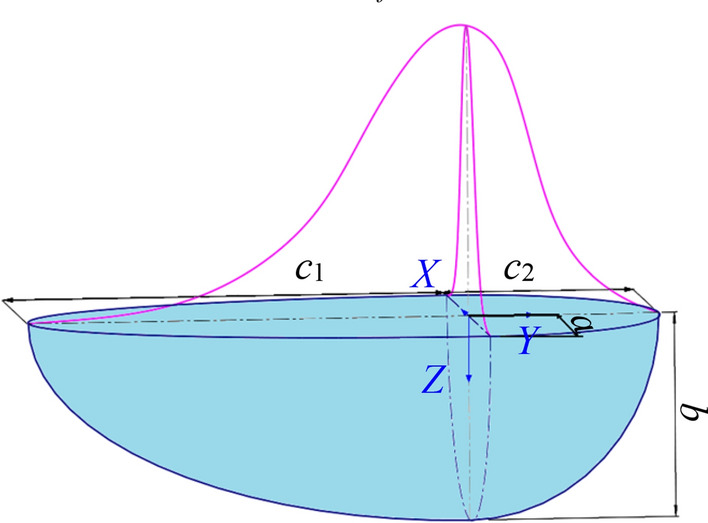
Gaussian double ellipsoid heat source model.

Gaussian double ellipsoid heat resource model shown in Fig. 4, is applied to describe the heat distribution of conventional MAG welding^27–30^. Set the half-axis parameters of the double ellipsoid to be $(a,b,c_{1} ,c_{2} )$, and the proportions of heat input in the front and back hemispheres are $f_{f}$ and $f_{r}$ respectively. $Q$ is the total heat input power.

The heat flow distribution of can be expressed by Formula (1, 2) as following^31^.

1 $$q_{f} (x,y,z) = \frac{{6\sqrt 3 f_{f} Q}}{{\pi \sqrt \pi abc_{1} }}\exp \left( { - \frac{{3x^{2} }}{{a^{2} }} - \frac{{3y^{2} }}{{c_{1}^{2} }} - \frac{{3z^{2} }}{{b^{2} }}} \right),\;\;y \ge 0$$

2 $$q_{r} (x,y,z) = \frac{{6\sqrt 3 f_{r} Q}}{{\pi \sqrt \pi abc_{2} }}\exp \left( { - \frac{{3x^{2} }}{{a^{2} }} - \frac{{3y^{2} }}{{c_{2}^{2} }} - \frac{{3z^{2} }}{{b^{2} }}} \right),\;\;y < 0$$

**Figure 5:**
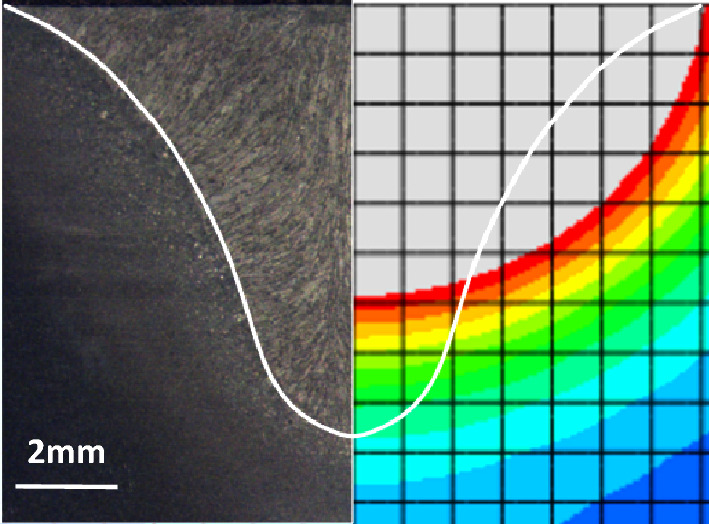
Simulation results of Gaussian double ellipsoid heat source.

Taking the fusion width as the end point of the iterative calculation, the fusion line is multiplied until $a = 14.35$, $b = 8.63$, $c_{1} = 6.3$, $c_{2} = 17.5$, $f_{f} = 0.4$, $f_{{\text{r}}} = 0.6$. Figure 5 illustrates the welding temperature distribution on the cross section of the workpiece, which was calculated using the double ellipsoid heat source model. The actual weld pool fusion line and upper surface boundary enclose an area of 54.42 mm^2^. However, the simulated fusion line and upper surface boundary enclose an area of 63.47 mm^2^, exhibiting a significant error of 16.63%. Moreover, the simulated contour morphology does not correspond to the actual shape of the flat upper and narrow lower surfaces.

Due to the significant difference between the high and low ends of Super Spray MAG joints, a combined heat source is being considered. To simulate the surface profile of the molten pool, a Gaussian heat source is utilized as the surface heat source. Additionally, a peak linear attenuation Gaussian cylinder heat source is used to simulate the penetration of the molten pool. The energy distribution coefficients are $\chi_{1}$, $\chi_{2}$ respectively.

Gauss surface heat source is used for the surface part of the workpiece, and the heat flux formula^32, 33^is

3 $$q(r,0) = \frac{{3\chi_{1} Q}}{{r_{TOP}^{2} }}\exp \left( { - \frac{{3r^{2} }}{{r_{TOP}^{2} }}} \right)$$

where $r_{TOP}$ is the effective action radius of surface Gaussian heat source. In order to reflect the energy distributing concentration of welding heat source. Define $r_{m}$ as the action radius when the welding heat source is not divergent (taking the inner diameter of the nozzle as the reference value), and $k$ is the ratio of the effective action radius $r_{TOP}$ and $r_{m}$,

4 $$k = {{r_{TOP} } \mathord{\left/ {\vphantom {{r_{TOP} } {r_{m} }}} \right. \kern-0pt} {r_{m} }}$$

where, replace Formula (3) with Formula (4) to obtain the final heat flux formula on the workpiece surface as following

5 $$q(r,0) = \frac{{3\chi_{1} Q}}{{\left( {kr_{m} } \right)^{2} }}\exp \left( { - \frac{{3r^{2} }}{{\left( {kr_{m} } \right)^{2} }}} \right) = \frac{{3\alpha \chi_{1} Q}}{{r_{m}^{2} }}\exp \left( { - \frac{{3\alpha r^{2} }}{{r_{m}^{2} }}} \right)$$

where, $\alpha$ is the heat flow concentration coefficient and defined as below

6 $$\alpha = \frac{1}{{k^{2} }} = \frac{1}{{\left( {{{r_{TOP} } \mathord{\left/ {\vphantom {{r_{TOP} } {r_{m} }}} \right. \kern-0pt} {r_{m} }}} \right)^{2} }}$$

The rest of the workpiece adopts cylinder heat source, described by following formula^34^,

7 $$q(r,z) = \frac{{3\chi_{2} Q}}{{\pi r_{0} H(2r_{0} - mH)}}\left( {1 - \frac{mz}{{r_{0} }}} \right)\exp \left( { - \frac{{3r^{2} }}{{r_{0}^{2} }}} \right)\;\;{\text{(z}} > {0)}$$

where, $r_{0}$ is the effective radius of the heat source, $H$ is the heat affected height, and $m$ is the heat flow attenuation factor.

**Figure 6:**
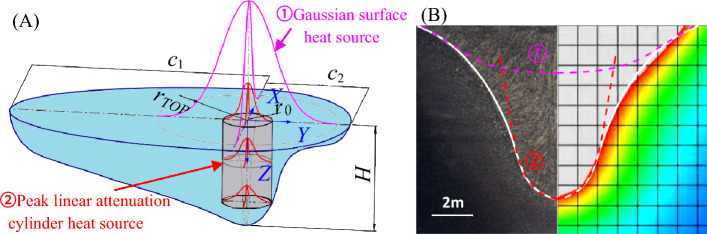
Gaussian surface heat source combined peak linear attenuation cylinder heat source: (**A**) heat transfer model, and (**B**) simulation results consistent with the actual measurement.

Taking the fusion width as the end point of iterative calculation^35^, the fusion line is multiply fitted until $r_{m}$ = 6.5, $\alpha$ = 1.65, H = 8.63, m = 0.2, $r_{0}$ = 1.95, $\chi_{1} = 0.3$, $\chi_{2} = 0.7$. The welding temperature field of workpiece cross section calculated by Combined heat source model is shown in Fig. 6. The area enclosed by the simulated fusion line and the upper surface boundary is 55.44 mm^2^, with a little error of 1.87%. The calculation accuracy is greatly improved, and the curve shape is in good agreement with the reality.

**Figure 7:**
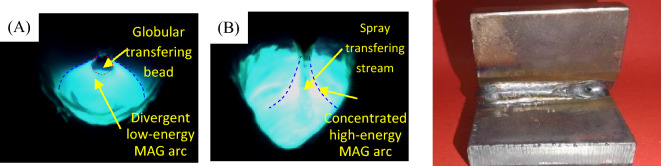
Comparison of welding arc and droplet transfer: (**A**) conventional MAG welding, (B) Super Spray MAG and (**C**) photograph of a welded joint.

The overall evaluation of Super Spray MAG arc energy concentration can be carried out as following. The total effective radius is $r_{TOP} = kr_{m} = \sqrt \alpha r_{m} = 8.35\;\;{\text{mm}}$, and 70% energy is concentrated within the effective action radius of cylinder heat source $r_{0} = 1.95\;\;{\text{mm}}$. The current density in this range is 77 times greater than the average current density, and the arc energy is efficiently compressed, exhibiting high-energy beam characteristics similar to those of lasers. Dynamic arc observations also verify and support this combined effect and high-energy beam characteristics. As shown in Fig. 7, in contrast to conventional MAG welding, the majority of the heat flux of Super Spray MAG arc is centered, indicating a reduced cylindrical heat source effect. However, there is still a certain level of conical divergence in arc compression, showing the Gaussian surface heat source effect^36^. Under different forms of heat source, the energy obtained from welding arc varies greatly. The droplet transfer in Super Spray MAG welding is primarily in the form of spray transfer, while conventional MAG welding typically employs globular transfer.

**Figure 8:**
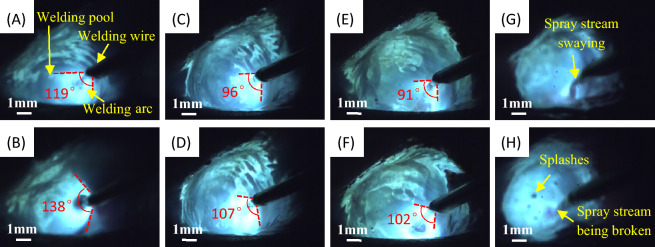
High speed photographs before and after arc jumping during Super Spray MAG welding at: (**A**, **B**) 280A welding current, 16 mm wire extension, continuous globular transfer; (**C**, **D**) 310A welding current, 16 mm wire extension, coarse flow spray transfer; (**E**, **F**) 340A welding current, 16 mm wire extension, trickle flow spray transfer with highly compressed arc; and (**G**, **H**) 310A welding current, 18 mm wire extension, unstable spray stream and splashes.

Further observation of welding arc and droplets was conducted by using high speed camera. The arc shape can directly reflect the degree of compression of the arc. As illustrated in Fig. 8A, B, at a welding current of 280 A and a wire extension of 16 mm, droplets are transferred in a globular shape and are wider than half the diameter of the welding wire. The arc exhibits divergence, instability, and oscillation, transitioning from an energy-dense cone to an energy-dispersive bell shape. Meanwhile, the droplet flow is intermittent and uneven. When the current is raised to 310 A and the wire extension is held at 16 mm, as depicted in Fig. 8C, D, droplets flow in a manner similar to a gurgling stream consisting of stream-like liquid beads. The arc has a tighter concentration with a smaller distribution angle and maintains its conical shape without noticeable changes. The droplets transfer streamly, uniformly and steadily, with a diameter of about half of the weld wire. When the welding current is increased to 340 A with a wire extension of 16 mm, there is a noticeable arc jump observed in Fig. 8E, F. However, the conical shape of the upper tip and lower flat remain intact, and the droplet spray stream becomes more uniform and coarse. Notably, when the wire extension was raised to 18 mm, as illustrated in Fig. 8G, H, the spray stream became unstable, resulting in breakage and splashing.

### Regularities of weld penetration

**Figure 9:**
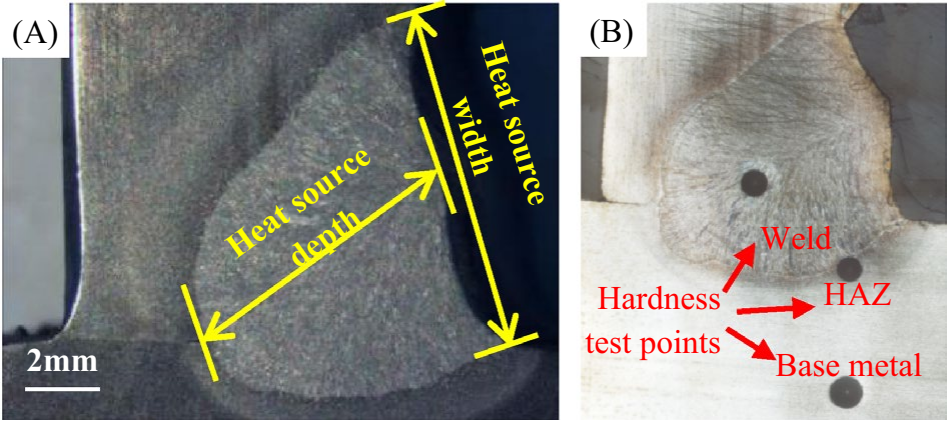
Measuring schematic diagram of weld cross section in (**A**) width and depth^37^, and (**B**) Brinell hardness.

For thick plates, narrow and deep cooled weld pools are expected. To analyze the regularities of arc control parameters on penetration, the depth and width of weld pools, i.e. heat source depth and heat source width, were inferred by intercepting the cross section as shown in Fig. 9. The depth-width ratio can characterize the penetration effectiveness of welding arcs.

The welding test procedure parameters and the weld cross section morphological data are shown in Tables 6, 7 and 8. According to the range analysis in the Heat source depth column of Table 8, the Range R values for welding current, wire extension, and welding speed were 4.48, 1.65, and 0.72, respectively. It can be concluded that the welding current mainly affects penetration, followed by wire extension. The welding speed has little effect on penetration. Increasing welding current within a certain range and reducing wire extension can effectively improve weld penetration and hardness. But after reaching a peak, weld penetration decreases with the increase of welding current. In contrast, on weld width, welding speed has the greatest influence, followed by wire extension, and welding current has no influence.

**Table 6:** Measurement results of T-joint weld formation in orthogonal welding tests.

Sample No	Welding current/A	Welding speed/(cm min^−1)^	Wire extension/mm	Heat source depth/mm	Heat source width/mm	Depth-width ratio	Brinell hardness/HB
1	250	30	10	8.06	7.04	1.14	228.51
2	250	35	12	7.84	7.22	1.09	222.60
3	250	40	14	7.22	7.30	0.99	220.18
4	250	45	16	7.04	7.46	0.94	226.72
5	250	50	18	6.88	7.58	0.91	232.18
6	280	30	12	10.08	7.64	1.32	212.41
7	280	35	14	9.77	7.86	1.24	207.58
8	280	40	16	8.75	9.42	0.93	211.27
9	280	45	18	7.88	10.39	0.76	237.38
10	280	50	10	10.38	8.32	1.25	228.02
11	310	30	14	12.68	9.26	1.37	204.25
12	310	35	16	10.42	10.81	0.96	203.41
13	310	40	18	10.55	11.04	0.96	203.5
14	310	45	10	13.22	8.96	1.48	218.91
15	310	50	12	12.56	9.80	1.28	217.45
16	340	30	16	11.52	10.82	1.06	202.63
17	340	35	18	10.87	10.91	1.00	203.98
18	340	40	10	11.76	10.50	1.12	198.28
19	340	45	12	11.64	10.58	1.10	201.75
20	340	50	14	11.38	10.72	1.06	202.34
21	370	30	18	10.82	11.38	0.95	186.55
22	370	35	10	11.84	10.66	1.11	175.84
23	370	40	12	11.26	10.94	1.03	172.36
24	370	45	14	10.96	11.06	0.99	168.52
25	370	50	16	11.12	11.32	0.98	178.23
26	280	35	16	10.72	8.92	1.20	203.2

**Table 7:** Measurement results of T-joint weld formation in random welding tests.

Sample no.	Welding current/A	Welding speed/(cm min^ −1)^	Wire extension/mm	Heat source depth/mm	Heat source width/mm	Depth-width ratio	Brinell hardness/HB
27	326	35	15	9.84	11.90	0.83	197.03
28	370	38	12	11.60	13.68	0.85	191.06
29	329	42	11	12.65	10.52	1.20	202.52
30	356	50	15	11.42	12.13	0.94	189.51
31	340	30	14	10.63	11.44	0.93	198.57
32	334	40	14	10.02	12.48	0.80	194.78
33	326	50	15	10.94	10.96	1.00	197.23
34	275	37	17	7.12	9.10	0.78	215.68
35	297	34	18	9.08	9.74	0.93	210.10
36	304	42	11	10.92	8.44	1.29	213.97
37	317	37	16	9.68	11.92	0.81	197.71
38	355	46	16	10.56	12.96	0.81	187.83
39	282	30	15	8.80	8.62	1.02	210.33
40	356	47	16	10.70	12.96	0.83	187.07
41	323	38	11	9.98	10.40	0.96	208.18
42	264	44	18	7.24	8.91	0.81	220.46
43	270	36	13	9.46	7.90	1.20	210.88
44	337	31	17	11.30	11.56	0.98	193.82
45	295	32	17	9.60	9.32	1.03	209.75
46	326	46	16	9.10	12.04	0.76	195.81

**Table 8:** Range analysis of measurement results of T-joint weld formation in orthogonal welding tests.

Index	Heat source depth/mm ↑			Heat source width/mm ↓			Depth-width ratio↑			Weld Brinell hardness/HB↑		
	A	B	C	A	B	C	A	B	C	A	B	C
Average k1	7.41	10.63	11.05	7.32	9.23	9.10	1.01	1.17	1.22	226.04	206.87	209.91
Average k2	9.37	10.15	10.68	8.73	9.49	9.24	1.10	1.08	1.16	219.33	202.68	205.31
Average k3	11.89	9.91	10.40	9.97	9.84	9.24	1.21	1.00	1.13	209.50	201.12	200.57
Average k4	11.43	10.15	9.77	10.71	9.69	9.97	1.07	1.05	0.98	201.80	210.66	204.45
Average k5	11.20	10.46	9.40	11.07	9.55	10.26	1.01	1.10	0.91	176.30	211.64	212.72
Range R	4.48	0.72	1.65	3.75	0.61	1.16	0.20	0.17	0.31	49.74	10.53	12.14
Preferred level	A3	B1	C1	A1	B1	C1	A3	B1	C1	A1	B5	C5
Order of actors	A > C > B			A > C > B			C > A > B			A > C > B		

### Microstructure evolution

**Figure 10:**
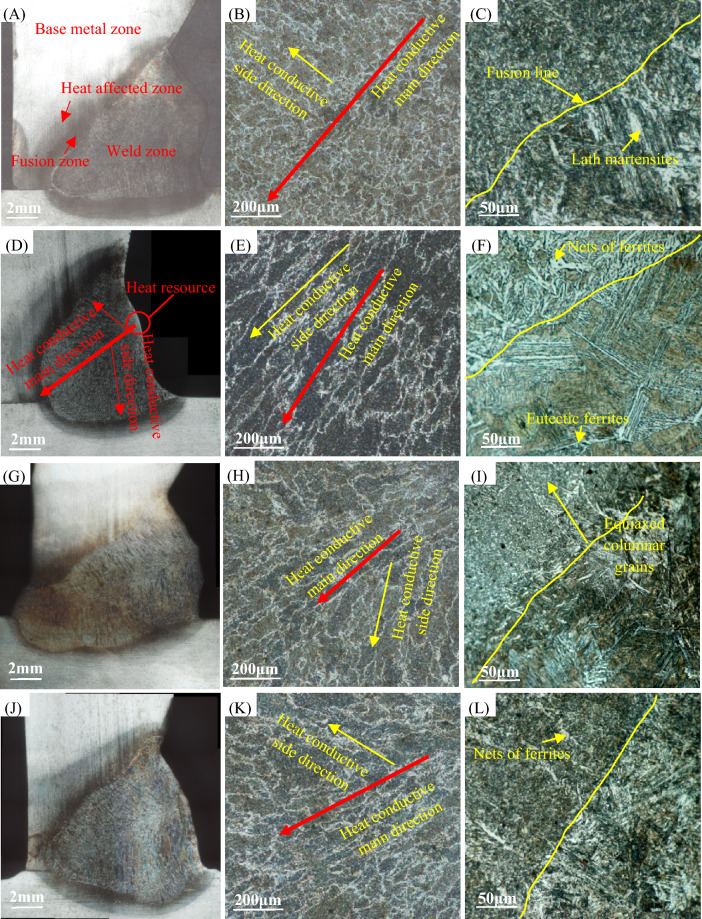
Microstructural morphology in the sequence of joints, weld zones, and areas adjacent to fusion lines: (**A**–**C**) sample 26 displays fine equiaxed grains in the central region, columnar crystals on branches, and lath martensites precipitated in slightly different directions in the fusion zone. (**D**–**F**) Sample 8 exhibits columnar crystals both on trunks and branches in the weld zone, and cross-bedded lath martensites in the fusion zone with wide and shallow penetration. (**G**–**I**) Sample 14, with full penetration, displays slender columnar crystals distributed like veins that extend to equiaxed grains at the ends of branches, revealing strong heat conductive apical dominance. (**J**–**L**) Sample 16 shows uneven columnar crystals with coarse grains scattering in the weld zone, and a certain amount of ferrite nets present beside the fusion lines even though it was incomplete penetration.

Analysis of grains and phases can assess how the microstructure evolves and whether the structure is reliable. It refers to Refs.^26, 38, 39^ to observe morphology. The microstructure morphology of some typical experimental weld joints is shown in Fig. 10. Each joint is divided into weld zone, fusion zone, heat affected zone and base metal zone. As mentioned in the literature, the weld zone is mainly composed of initial austenite grains and net pre-eutectic ferrites precipitated between grains. The fusion zone has the deepest color and forms an obvious fusion line with the weld zone, where cracks are most likely initiated. The heat affected zone is lighter than the fusion zone and darker than the base metal. The base metal shows typical rolling flow lines, with grain flattened and elongated and black pearlites scattered between the flow lines.

In typical MAG welding joints, depicted in Fig. 10A–C, the central area displays fine equiaxed grains, with more well-formed columnar crystals in the branching direction. The heat affected zones exhibit strip martensites with slight variations in orientation. But for Super Spray MAG welding, as shown in Fig. 10D–L, apical dominance of heat conduction is more evident. The austenite grains are radially distributed symmetrically from the center like veins on a leaf. This suggests that heat conduction from the center of the heat source is most efficient, spreading along the trunk and then outwards along the lateral branches during welding. With the change of arc control parameters, the weld penetration of all samples changes greatly and the precipitated structure is similar. But its distribution and morphology, especially on both sides of the fusion line, are very different. Based on the microstructure morphology of the welded zone and surrounding regions, it can be observed that, under low current of 280 A, the initial austenite grains in the weld zone appear to be coarse. Additionally, the pre-eutectoid ferrite grids display uneven patterns with wide boundaries. There are few granular pearlites and granular ferrites precipitated in the crystal. In the fusion zone, strip martensites precipitated between pearlites and ferrites. As the welding current increases, the austenite grains in the weld zone become more refined, and the pre-eutectoid ferrite grids become shorter and slender. The precipitation of pearlites and ferrites occurs more appropriately in crystals, and martensites in the fusion zone gradually change from a regular strip shape to a more interlaced flake or pine needle shape. Of all samples, Sample 14 was fully penetrated with the most developed trunk and shortest branches. There is a tendency for initial austenite crystals to become equiaxed at the ends of branches where pre-eutectoid ferrites are present in lower amounts and pearlites are precipitated more frequently. Therefore, the heat transfer in the penetration direction is faster and further than that in the width direction.

### Optimization of welding process parameters

**Figure 11:**
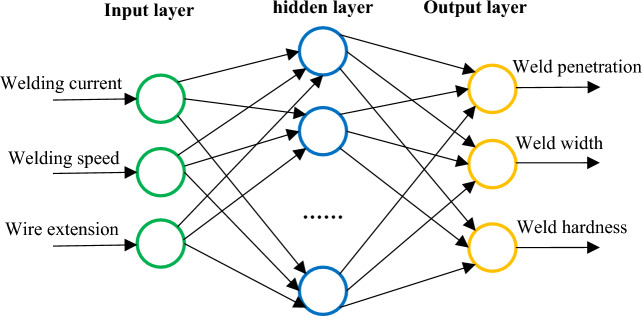
Back Propagation Neural Network (BPNN) model.

Back Propagation Neural Network (BPNN) model for Super Spray MAG Welding were specified in Fig. 11. The input layer had 3 nodes, i.e. welding current, welding speed and wire extension. The output layer also had 3 nodes, weld penetration, weld width and weld hardness. Using the data in Tables 6 and 7, 70% were selected for training, 15% for verification, and 15% for simulation. Through repeated tests, 12 were selected as the number of hidden layer nodes under the condition that the network error is as small as possible.

**Figure 12:**
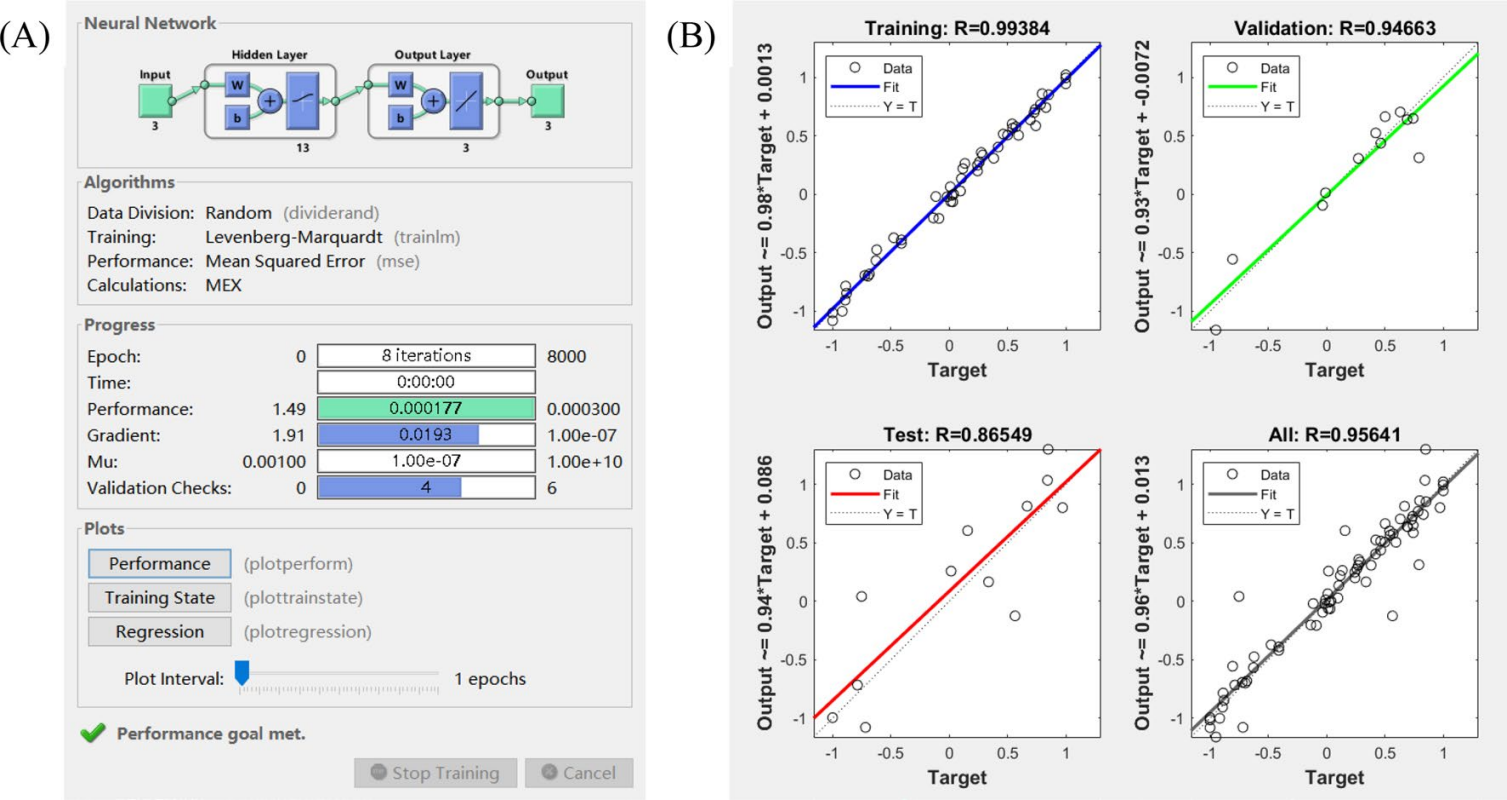
Neural network (**A**) training results, and (**B**) prediction confidence.

The transfer function between the input layer and the hidden layer was set to function *tansi*g, the transfer function between the hidden layer and the output layer was function *purelin*, and the network training function is function *trainlm*. Due to the large difference in the range of factors, function *mapminmax* was used to normalize the input parameters, and the output parameters were anti-normalized. The max-epoch for the training network was 8000, and the mean square deviation was below 0.0003. The MATLAB program code for building neural networks was as follows:
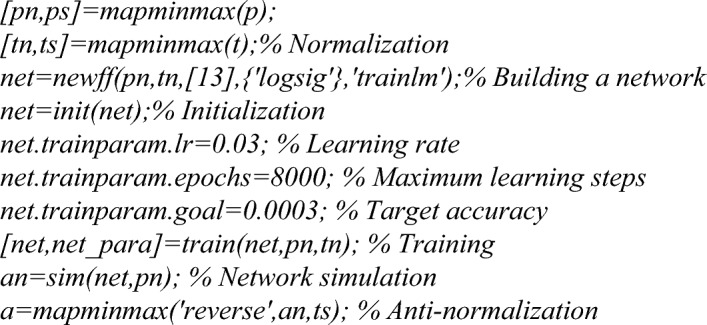


After 8 iterations, the target accuracy requirements were met as shown in Fig. 12A. Although the BP neural network had been formed and the corresponding parameters of each layer had been obtained, model verification was required. The accuracy and generalization of the model were fully verified with the remaining 8 groups of experimental data. Figure 12B shows the audit results. It can be seen that the confidence levels of the training, verification and test samples were all above 85%.

**Figure 13:**
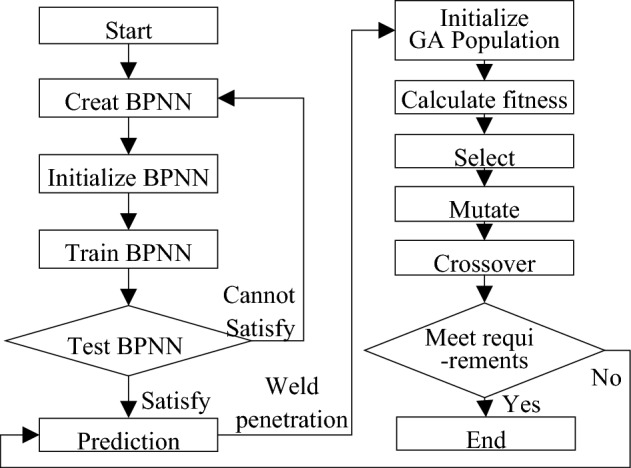
Flow chart of Genetic Algorithm based on BPNN.

Then, based on BPNN, Genetic Algorithm (GA) was performed for optimization, as shown in Fig. 13. BPNN prediction data were imported into the GA model for fitness calculation, and individuals with lower fitness values were selected as breeding parents. Then, the propagated sub-individuals were converted into new prediction data, simulated by neural networks and calculated fitness. This cycle continued until the optimal individual was finally obtained.

The weld penetration was calculated by using the neural network constructed by calling the fitness function, and the fitness value was obtained by comparing the weld penetration with the target value of 12 mm. The construction program code is as follows:
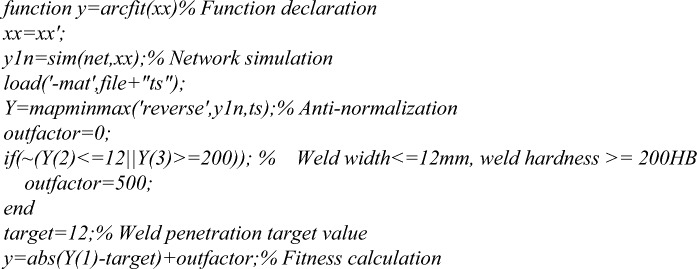


Call function *Genetic Algorithm* in MATLAB for multi-objective optimization, take the number of variables nvars = 3, the lower limit of variables lb = [280 30 10], the upper limit ub = [370 50 18]. The MATLAB function program code is as follows:
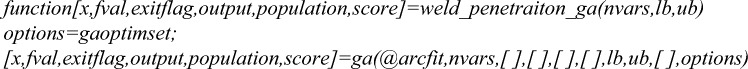


Through calculation, the optimal solution is 316.0877, 42.5068, 12.1823. The rounding parameters are: welding current 316 A, welding speed 42 mm/min, and wire extension 12 mm.

### Verification

The optimum parameters are substituted in the BPNN model for simulation verification, and the weld penetration is 12 mm, the weld width is 9.34 mm, the Brinell hardness of the weld is 224.23 HB, and the error is less than 5%. The precise control of welding formation and mechanical properties is realized, and the accuracy of genetic algorithm optimization is verified.

Moreover, the results of tensile, bending and impact tests are shown in Table 9, indicating that tensile strength of all joints is greater than that of the base metal. The fracture position of the tensile sample is not at the weld seam but in the base metal zones, which can meet the design requirements. There are no defects on the surface of the four bending samples, and the plasticity of the welded joint is perfect. The impact energy of weld and heat affected zone at – 20 ℃ is higher than that of the base metal. Ultrasonic flaw detection was carried out to detect internal defects. The Grade I qualification rate for ultrasonic flaw detection of the preferred combination is over 90%.

**Table 9:** Mechanical test data of optimized combination.

Index	Tensile strength/MPa	Bending test	Impact test/J		UT flaw detection qualification rate of Grade I
			Weld zone	Heat affected zone	
Test values	535、537	No defects	259.5, 254, 244.5	163.5, 254.5, 139.5	100%
Required values	≥ 470	surface breaking length < 3 mm	≥ 47	≥ 47	> 90%

### Application

**Figure 14:**
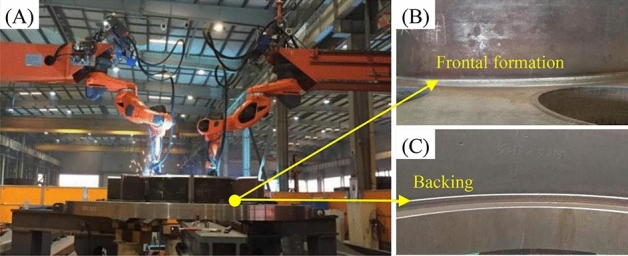
Application of Super Spray MAG welding to automatic welding of gearboxes of TBMs: (**A**) welding of gearbox composed of 60–140 mm thick plates with diameter of 4.8 m, (**B**) perfect frontal formation, and (**C**) perfect backing formation.

**Figure 15:**
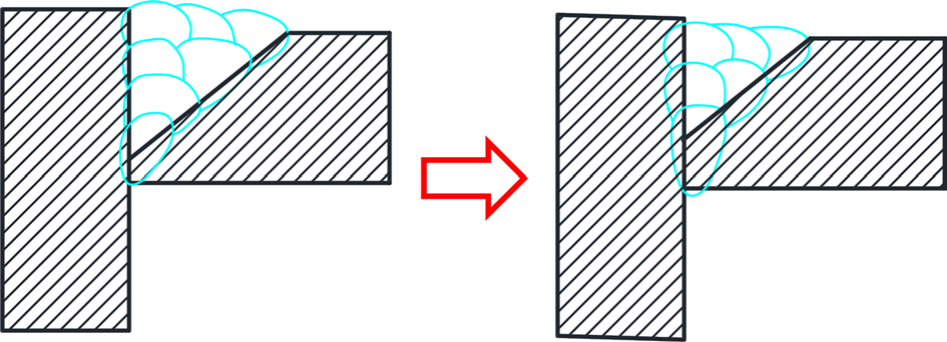
Filling metal reducing diagram by groove optimization.

Deep penetration technology, using Super Spray MAG welding, has been successfully applied to weld TBM main drives, cutterheads, and various structural components, as depicted in Fig. 14. Moreover, the initial pass rate for Grade I ultrasonic flaw detection of welded joints exceeds 99%, enabling complete welding automation for critical TBM components. Furthermore, under the requirements of equal strength, as shown in Fig. 15, Super Spray MAG welding could reduce the volume of groove cutting and require less filler metal, saving energy and material. Generally, the amount of welding wire used and the energy consumption of Super Spray MAG Welding can be reduced by 25%, respectively, and the direct cost can be reduced by 30% compared to conventional MAG welding. It not only greatly improves weld quality and welding efficiency, but also becomes more green and environmentally friendly.

## Conclusion

The heat source properties, penetration mechanism, and valuable processing parameters for Super Spray MAG welding were investigated. Compared to traditional GMAW welding, eliminating pulses and achieving a consistent and dependable spray welding process can greatly enhance welding efficiency and achieve full penetration.

- The heat flux of Super Spray MAG welding can be described by the combined model of Gaussian surface heat source and peak linear attenuation Gaussian cylinder heat source. The simulation results are highly consistent with the actual shape of the molten pool.
- Weld penetration is very sensitive to changes in welding current and wire extension. Increasing welding current within a certain range and reducing wire extension can effectively improve weld penetration, strength, hardness and qualification rate for defect detection. But after reaching a peak, weld penetration and joint performance decline as welding current increases.
- Super Spray MAG arc has a high energy concentrated heat flux, resulting in higher pool temperature, stronger diffusion and faster nucleation than conventional MAG welding. In a certain range, welding current can improve pearlite precipitation and refinement of pre-eutectoid ferrites in the weld zone, and promote martensite refinement.
- The BPNN-GA model is capable of accurately predicting weld formation and facilitating progress optimization. For achieving a 5 mm penetration with Super Spray MAG welding, recommended parameters include a welding current of around 316 A, a welding speed of 42 cm/min, and a wire extension of 12 mm.

At present, the Super Spray MAG welding equipment still has a heavy and complex structure, and needs simplification to improve the accessibility of all positions. This will enable the full automation of welding for even more multifaceted structural components.

## Data Availability

In relation to the availability of raw data I want to inform you that this work is a part of a long research study, and the raw data will not be public at the moment but are available from corresponding author upon the request.
